# Diagnostic value of endothelial-specific molecule-1 levels in patients with bone tumors

**DOI:** 10.3389/abp.2026.17417

**Published:** 2026-09-10

**Authors:** Eyup Senocak, Nurinnisa Ozturk, Adem Keskin, Umit Aygun, Ibrahim Dag, Muhammet Celik

**Affiliations:** 1 Department of Orthopedics and Traumatology, Faculty of Medicine, Ataturk University, Erzurum, Türkiye; 2 Department of Medical Biochemistry, Faculty of Medicine, Ataturk University, Erzurum, Türkiye; 3 Department of Medical Biochemistry, Faculty of Medicine, Aydin Adnan Menderes University, Aydın, Türkiye; 4 Department of Orthopedics and Traumatology, Faculty of Medicine, Agri Ibrahim Cecen University, Agri, Türkiye

**Keywords:** bone tumor, diagnostic value, ESM-1, malignancy, sensitivity

## Abstract

**Background:**

In suspected bone tumors where imaging and histopathological evaluation are fundamental for diagnosis, minimally invasive blood biomarkers are needed for earlier diagnosis, initial assessment, and risk stratification. The aim of this research was to evaluate the diagnostic value of Endothelial-Specific Molecule-1 (ESM-1) levels in determining the presence of bone tumors and distinguishing between benign and malignant tumors.

**Methods:**

This cross-sectional research, conducted at a research hospital, involved 60 participants diagnosed with bone tumors and 30 healthy participants. ESM-1 values were analyzed using Enzyme-Linked Immunosorbent Assay (ELISA). The data obtained were evaluated using binary logistic regression analysis and Receiver Operating Characteristic (ROC) curve analysis.

**Results:**

ESM-1 values were determined to be higher in the bone tumors group than in the control group. ROC analysis showed an Area Under the Curve (AUC) of 0.895. Using a cut-off value of 138.94 ng/L for detecting bone tumors, the sensitivity and specificity were 78.3% and 80.0%, respectively. In addition, ESM-1, erythrocyte sedimentation rate, and C-reactive protein levels were found to be higher in the malignant subgroup than in the benign subgroup. In subgroup ROC analysis, ESM-1 perfectly discriminated against malignant tumors from both benign tumors (AUC = 1.000) and healthy controls (AUC = 1.000), with sensitivities and specificities of 100% at cut-off values of 204.54 ng/L and 195.00 ng/L, respectively. For differentiating benign tumors from healthy controls, the AUC was 0.789, with 70.0% sensitivity and 70.0% specificity at a cut-off value of 125.54 ng/L.

**Conclusion:**

ESM-1 levels may have potential diagnostic value in distinguishing between bone tumors and malignant tumors; however, these results need to be confirmed in larger samples.

## Introduction

Despite their rarity, bone tumors have significant impacts on patient health and treatment outcomes. These tumors represent an important area of oncology due to their unique epidemiological characteristics and clinical impact. Because survival rates are higher in localized diseases compared to metastatic cases, early diagnosis and treatment are critical. Survival rates vary among tumor types highlight the necessity for personalized therapy approaches. While advances in imaging modalities and in-creased clinical awareness have increased early diagnosis rates, research is still needed to improve diagnostic accuracy and treatment approaches (Hosseini et al., 2025). The observed changes in the epidemiology of bone tumors are thought to be related to many factors, including genetic predisposition, environmental factors, lifestyle, and access to healthcare. Improved knowledge of these interactions will support both clarifying the causes of the disease and improving diagnostic, therapeutic, and monitoring processes. Therefore, comprehensive research is needed to elucidate epidemiological transitions and underlying causes (Xu et al., 2024).

Malignant bone tumors are clinically extremely diverse and difficult to diagnose due to their rarity and nonspecific symptoms, which often delay diagnosis. Compared to the previous few decades, the prevalence of these tumors has increased in the past decade, and mortality and survival rates vary among subgroups depending on factors such as age and tumor type (Xu et al., 2024). Furthermore, because late diagnosis of primary malignant bone tumors leads to poor prognosis, these tumors should not be overlooked in routine clinical practice and should be considered in the differential diagnosis of musculoskeletal complaints (Funovics, 2023). On the other hand, benign cases are far more common than malignant cases and are frequently observed in daily clinical care. Imaging plays a crucial role in determining the non-aggressive nature of these lesions and assessing the need for further imaging or follow-up (Naraghi et al., 2022).

Endothelial-Specific Molecule-1 (ESM-1), produced by endothelial cells, is a soluble proteoglycan. Increased ESM-1 levels have been shown in both tumor cells and tumor feeding vessels in various human cancers. High ESM-1 levels are associated with poor prognosis in many cancers (Reikvam et al., 2022). Furthermore, increasing evidence suggests that ESM-1 facilitates tumor progression by enhancing cancer cell motility, proliferation, and cancer stem cell properties. Furthermore, several proteins that interact with ESM-1 in cancer cells and different subcellular localizations of ESM-1 have recently been identified (Pan et al., 2022). ESM-1 is also considered a potential immune inflammatory marker (Chen et al., 2022). In addition to all these, ESM-1 has become the focus of many studies in recent years due to its relationship with many diseases, from neurological diseases to cardio metabolic disorders, from lung, kidney and liver diseases to cancer (Reikvam et al., 2022; Pan et al., 2022; Chen et al., 2022; Liu et al., 2024; Klisic and Patoulias, 2023; Behnoush et al., 2024; Khalaji et al., 2023; Samouilidou et al., 2022; Klisić et al., 2020).

Despite their rarity, bone tumors have significant impacts on patient health and treatment outcomes. Imaging and histopathological evaluation remain key methods for diagnosis and risk assessment. However, these approaches are largely dependent on the morphological changes caused by the tumor, which can limit assessment in the early stages of the disease. Therefore, there is a need for minimally invasive and easily applicable blood-based biomarkers that can provide complementary information about the presence and biological behavior of bone tumors before significant morphological changes occur. Early diagnosis and reliable risk stratification can facilitate timely referral of appropriate patients and support improved treatment outcomes.

ESM-1, also known as endocan, is a circulating proteoglycan produced primarily by endothelial cells and involved in endothelial activation and angiogenesis; these biological processes may be relevant to tumor development and progression. These biological properties justify investigating ESM-1 as a potential blood-based biomarker in bone tumors. However, despite this biological rationale, the diagnostic value of serum ESM-1 in bone tumors has not been sufficiently characterized. Specifically, it is unclear whether ESM-1 can differentiate patients with bone tumors from healthy individuals, and whether it can distinguish malignant bone tumors from benign bone tumors.

Therefore, this study aimed to evaluate the diagnostic value of serum ESM-1 not only in detecting the presence of bone tumors but also in differentiating between benign and malignant tumors. By addressing this gap in the current literature, this study may contribute to the evaluation of ESM-1 as a minimally invasive blood-based biomarker that will complement current diagnostic approaches and support early diagnosis and risk stratification in patients with bone tumors.

## Materials and methods

### Study population and eligibility criteria

The sample size required for the study was calculated using the G*Power 3.1.9.7 power analysis software. The analysis was performed under the following parameters: test family: t tests; statistical test: means – difference between two independent means (two groups); type of power analysis: *a priori* (compute required sample size given α, power, and effect size). An effect size (d) of 0.5, α = 0.05, power (1−β) = 0.95, and an allocation ratio (N_2_/N_1_) = 2 were used. Considering this result; Sixty participants diagnosed with bone tumors who presented to the Orthopedics Polyclinic of Atatürk University Research Hospital between 1 August 2023, and 31 December 2024, were involved in the research as the bone tumor group, while 30 healthy individuals were included as the control group. To ensure a balanced composition of the subgroups, care was taken to ensure that the bone tumor group consisted of an equal number of benign and malignant cases.

Inclusion criteria included participants with primary malignant tumors and participants with bone metastases in the malignant subgroup. The benign subgroup included patients with biopsy confirmed benign bone tumors. All malignant bone tumor cases, including primary malignant tumors and bone metastases, were confirmed histopathologically. The control group comprised healthy participants without a history of orthopedic or systemic malignancy or benign tumor and with similar gender and age characteristics as the bone tumor group. Participants undergoing active therapy for their primary malignant illness or receiving radiotherapy or/and chemotherapy after metastasis weren’t included. Additionally, individuals with a BMI over 30 kg/m^2^, systemic diseases such as metabolic syndrome, hyperlipidemia, hypertension, coronary artery disease, hepatic or renal insufficiency, or body’s defences system disorders were excluded. Individuals with no definitive pathological diagnosis and only radiological benign tumors were also excluded.

### Biochemical measurements

Venous blood specimens were obtained from participants in the groups. Whole blood specimens were analyzed for complete blood count using a XN-1000 Analyzer (Sysmex Corp., Kobe, Japan). For biochemistry analysis, blood specimens were transferred to appropriate tubes and centrifuged at 3,000 rpm. Serum specimens were kept at −80 °C until ESM-1 levels were measured.

Once enough specimens were collected, the specimens were removed from the freezer and thawed at room temperature and ESM-1 values of all specimens were analyzed simultaneously. Measurements were performed using the ESM-1 ELISA Kit (Bioassay Technology Laboratory, Catalog Number: E3160Hu, Zhejiang, China) and Dynex ELISA instrument (Dynex Technologies, Chantilly, VA, USA). All specimens were analysed in duplicate.

### Statistical analysis

Statistical tests were performed via IBM SPSS Statistics 22.0 software. Continuous variables were presented as mean ± standard deviation and categorical data as percentage and number. The Mann–Whitney U test was used for continuous data, and the chi-square test was used for categorical data. Spearman correlation test was performed to see the relationship between ESM-1 levels and other laboratory findings. Receiver Operating Characteristic (ROC) and binary logistic regression tests were applied for ESM-1 levels. The optimal cut-off value was determined based on the maximum Youden index (J = sensitivity + specificity-1).

## Results

### Comparison of demographic and laboratory findings of bone tumor and control groups

The average age of the bone tumor group was 48.45 ± 19.8 years, while the mean age of the control group was 49.93 ± 16.79 years. Additionally, 30 (50%) of the bone tumor group were female, and 15 (50%) of the control group were female. There was no important difference between the two groups in terms of gender ratio and average age (p = 1.000, p = 0.691, respectively). Laboratory parameters of these groups are demonstrated in Table 1.

**TABLE 1:** The laboratory parameters of groups.

Parameters	Control group (n = 30)	Bone tumor group (n = 60)	P
ESM-1 (ng/L)	103.8 ± 38.66	246.63 ± 111.56	<0.001
WBC (×10^9^/L)	8.72 ± 2.25	8.19 ± 2.38	0.532
NLR	2.7 ± 1.84	4.22 ± 6.45	0.687
Hemoglobin (g/dL)	12.23 ± 2	12.87 ± 2.11	0.173
CRP (mg/L)	-	14.60 ± 30.89	-
ESR (mm/h)	-	20.78 ± 21.24	-

WBC: white blood cell count, ESR: erythrocyte sedimentation rate, NLR: Neutrophil-to-Lymphocyte Ratio, CRP: C-Reactive Protein.

ESM-1 values were higher in the bone tumor group than in the control group (Table 1).

### Diagnostic performance of endocan levels for bone tumor presence

Binary logistic regression analysis was tested to determine the predictive power of ESM-1 levels for bone tumor presence. As a result of the analysis, the accuracy of ESM-1 levels in predicting tumor presence was determined as 78.9%. In the model, ESM-1 level was found to be a significant variable in terms of predicting bone tumor presence (p < 0.001). Odds ratio was 1.038.

**FIGURE 1:**
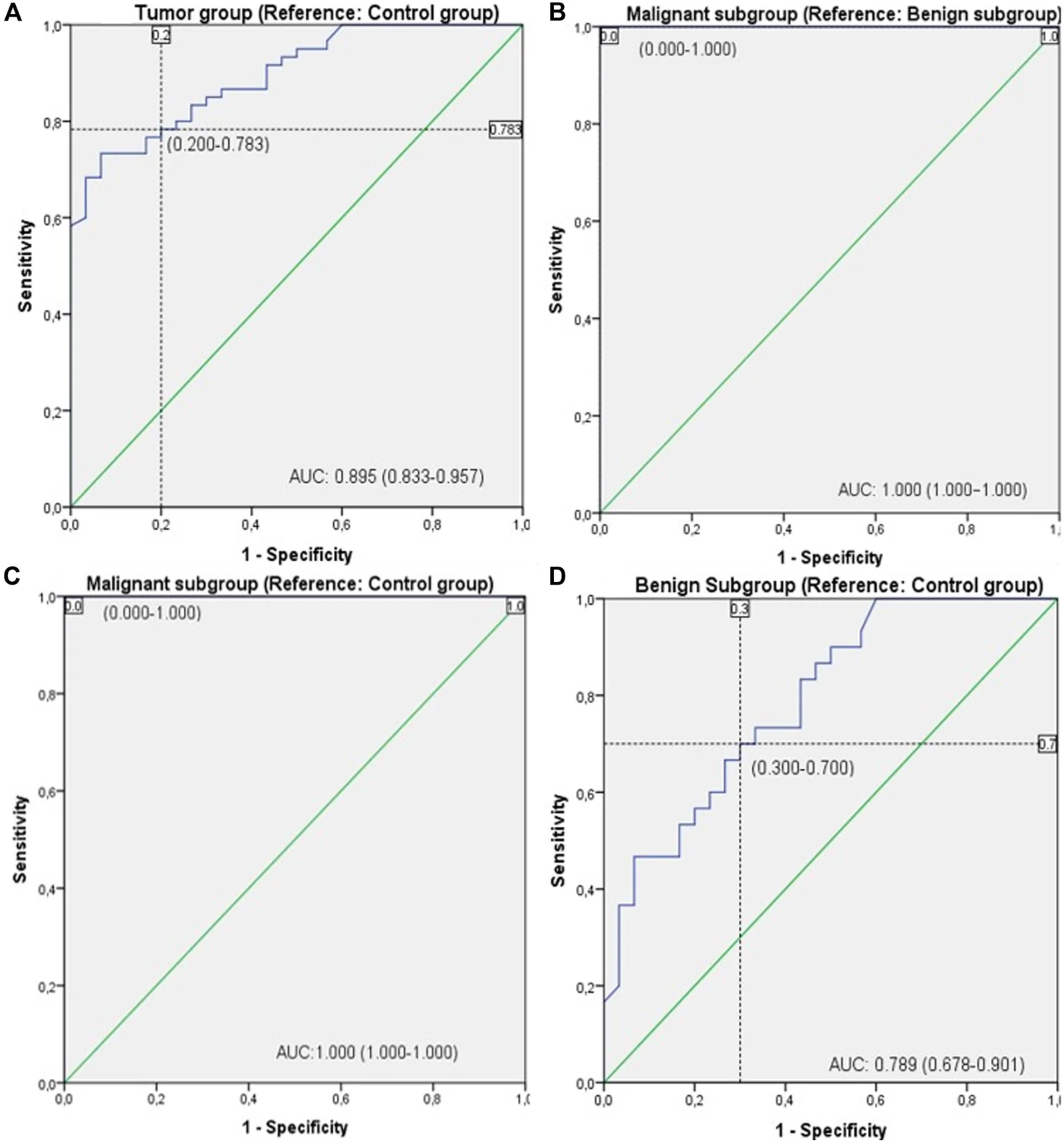
ROC Curve Graphs of bone tumor group and its subgroup.

ROC curve analysis was tested to determine the specificity, sensitivity and cut-off value of ESM-1 values in predicting bone tumor presence (Figure 1A). In the ROC and logistic regression analyses, the control group was designated as the reference category to evaluate the discriminative power of ESM-1 values in predicting bone tumor presence. The results of the ROC curve analysis are shown in Table 2.

**TABLE 2:** ROC analysis results of ESM-1 values to predict the presence of bone tumors.

Area under curve (95% confidence interval)	0.895 (0.833–0.957)
Cut-off	138.94
P	<0.001
Sensitivity (%) (95% confidence interval)	78.3 (66.4–86.9)
Specificity (%) (95% confidence interval)	80 (62.7–90.5)
Positive predictive value (%) (95% confidence interval)	88.70 (80.20–97.20)
Negative predictive value (%) (95% confidence interval)	64.80 (49.40–80.20)

### Comparison of demographic and laboratory findings of benign and malignant bone tumor subgroups

It was determined that 30 (50%) of the patients in the tumor group had benign tumors, while 30 (50%) had malignant tumors. Accordingly, two subgroups were formed in the tumor group: benign and malignant. The average age of the malignant subgroup was 50.33 ± 21.29 years, while the average age of the benign subgroup was 46.57 ± 18.43 years. In addition, 14 (46.67%) of the malignant subgroup were female and 16 (53.33%) of the control group were female. There was no important difference between the two subgroups in terms of gender ratio and average age (p = 0.606, p = 0. 478, respectively). Laboratory parameters of benign and malignant subgroups are presented in Table 3.

**TABLE 3:** The laboratory parameters of subgroups.

Parameters	Benign subgroup n = 30	Malignant subgroup n = 30	P
ESM-1 (ng/L)	142.63 ± 28.05	350.62 ± 46.46	<0.001
WBC (×10^9^/L)	8.19 ± 2.38	9.07 ± 4.30	0.641
NLR	2.57 ± 1.44	5.87 ± 8.78	0.344
Haemoglobin (g/dL)	13.09 ± 2.33	12.66 ± 1.88	0.451
CRP (mg/L)	3.63 ± 5.93	25.36 ± 41.39	<0.001
ESR (mm/h)	9.69 ± 14.79	31.87 ± 21.26	<0.001

WBC: white blood cell count, CRP: C-Reactive Protein, NLR: Neutrophil-to-Lymphocyte Ratio, ESR: erythrocyte sedimentation rate.

The mean levels of ESM-1, C-Reactive Protein (CRP), and erythrocyte sedimentation rate (ESR) were determined to be higher in the malignant subgroup than in the benign subgroup (Table 3). Correlation analysis was tested to examine the relation between ESM-1 values and CRP, and ESR levels. ESM-1 levels were determined to be positively correlated with CRP (correlation coefficient (r): 0.570, p < 0.001) and ESR (r: 0.561, p < 0.001).

### Diagnostic performance of endocan levels in predicting bone tumor malignancy or benignity

Binary logistic regression analysis was performed to determine the predictive power of ESM-1 levels for the presence of malignancy. As a result of the analysis, the accuracy of ESM-1 levels in predicting for the presence of malignancy was determined as 100%. In the model, the ESM-1 level was found to be a significant variable in terms of predicting presence of malignancy (p < 0.001). Odds ratio was 2.759.

ROC curve analysis was tested to determine the specificity, sensitivity, and cut-off value of ESM-1 values in predicting the presence of malignancy (Figure 1B). The benign subgroup data were used as the reference to assess the ability of ESM-1 levels to distinguish between malignant and benign bone tumors. Additionally, ROC analyses were tested to determine the specificity, sensitivity, and cut-off value of ESM-1 levels according to malignant or benign status, using control group data as reference (Figures 1C,D respectively). The results of the analysis are shown in Table 4.

**TABLE 4:** ROC analysis results of ESM-1 levels in distinguishing benign and malignant bone tumors.

Subgroup	Malignant	Malignant	Benign
Reference	Benign	Control	Control
AUC (95% CI)	1.000 (1.000–1.000)	1.000 (1.000–1.000)	0.789 (0.678–0.901)
Cut-off	204.54	195.00	125.54
P	<0.001	<0.001	<0.001
Sensitivity (%) (95% CI)	100 (88.4–100)	100 (88.4–100)	70 (50.6–85.3)
Specificity (%) (95% CI)	100 (88.4–100)	100 (88.4–100)	70 (50.6–85.3)
PPV (%) (95% CI)	100 (88.4–100)	100 (88.4–100)	70 (50.6–85.3)
NPV (%) (95% CI)	100 (88.4–100)	100 (88.4–100)	70 (50.6–85.3)

AUC: area under curve, CI: Confidence interval PPV: positive predictive value, NPV: negative predictive value.

ESM-1 demonstrated excellent predictive performance for identifying malignant bone tumors, with both PPV and NPV reaching 100% (95% CI: 88.4–100) when malignant cases were compared with either benign tumors or healthy controls. However, its predictive performance for differentiating benign bone tumors from healthy controls was lower, with both PPV and NPV of 70.0% (95% CI: 50.60–85.30).

## Discussion

It is known that ESM-1 increases the effect of receptor tyrosine kinases in tumor formation. It is also a key regulator of the epidermal growth factor/epidermal growth factor receptor signalling pathway. Therefore, ESM-1 levels have been found to be higher in lung tumors compared to non-cancerous tissues (Yang et al., 2020). Similarly, in a recent case-control study, ESM-1 values were determined to be higher in individuals with bladder cancer compared to healthy volunteers. Additionally, in ROC analysis, sensitivity was reported as 59.1%, specificity as 57.7%, and AUC as 0.609 (Okucu et al., 2023). In another study examining both *in vitro* cell proliferation and *in vivo* tumor growth, ESM-1 expression was determined to be markedly increased in highly aggressive triple negative breast cancers compared to less aggressive subtypes. In addition, it has also been reported that the prognosis of triple negative breast cancer patients can be easily predicted by measuring plasma ESM-1 levels through simple blood sampling (Sagara et al., 2017).

In this study, bone cancer patients were found to have higher ESM-1 levels than healthy participants. In ROC analysis, the AUC value was 0.895 with a sensitivity of 78.3% and specificity of 80%.

ESM-1, which is associated with abnormal differentiation and high invasive capacity in stomach cancer, plays a role in numerous cellular proliferation processes. ESM-1 plays a role in various processes such as cell proliferation, migration, and tumor progression, and due to these characteristics, it is considered a potential target protein in anticancer research (Zhong et al., 2026). A recent study investigating novel biomarkers for colorectal cancer through intestinal tissue profiling using the olink method reported that among 68 proteins differentially expressed between tumor and normal tissues, ESM-1 was one of three biomarkers showing relatively strong correlations and exhibiting the most significant changes compared to other markers (Xiao et al., 2025). A recent study involving patients diagnosed with renal cell carcinoma (RCC) and with an average follow-up period of 71 months reported that immunohistochemical expression of ESM-1 may be a promising prognostic biomarker in RCC (Bocu et al., 2023). This finding suggests that ESM-1 may have potential clinical value not only diagnostically but also in assessing disease prognosis in various malignancies.

Metastasis is an important cause of morbidity and mortality in cancer patients. The molecular mechanisms by which metastatic cancer recognizes, infiltrates, and colonizes bone are not yet fully understood (Coniglio, 2018). The most common malignancies, such as prostate, breast, and lung cancer, frequently metastasize to bone. Bone metastases are a significant cause of morbidity in these patients and are related with poor prognosis. The skeletal system is a preferred site of metastasis for these solid tumors, and bone metastases can occur in up to 70% of patients with advanced breast or prostate cancer (Guise, 2010). In contrast, the endothelium plays a crucial role in cancer metastasis. Endothelial inflammation, glycocalyx disruption, and elevated endothelial permeability are key events in early metastasis. Furthermore, levels of ESM-1, one of the molecules associated with endothelial function, appear to increase in the early stages of metastasis (Suraj et al., 2019).

In a study following prostate cancer patients for up to 5 years, high ESM-1 values were reported to be an important predictor of progression free survival. It has also been reported that ESM-1 has a close relationship with tumor recurrence in prostate cancer (Arslan et al., 2017). In a study conducted on patients with endometrial and ovarian carcinoma, the two most common malignancies of the female reproductive system, it was reported that benign ovarian or endometrial disorders did not cause a rise in ESM-1 expression, but that there was a rise in ESM-1 expression in malignant cases. Additionally, a positive correlation was found between CA-125 and ESM-1 levels in all patients, and it was reported that ESM-1 levels may be useful in distinguishing between benign and malignant endometrial or ovarian diseases (Laloglu et al., 2017). On the other hand, in a study conducted on breast tissue, ESM-1 levels were compared between inflammatory and malignant diseases; it was reported that ESM-1 levels were higher in individuals with malignant diseases than in healthy individuals, but there was no significant difference in those with inflammatory diseases (Basim and Argun, 2021).

In this research comparing ESM-1 values in patients with malignant and benign bone tumors, both malignant and benign participants had higher ESM-1 values than healthy individuals. Furthermore, ESM-1 levels were higher in malignant participants than in benign participants. In ROC analyses using both healthy individuals and benign patients as references to predict malignant tumors, the AUC value was 1.000, sensitivity was 100%, and specificity was 100%. In addition, in the ROC analysis performed to distinguish benign patients from healthy individuals, the AUC value was found to be 0.789, sensitivity was 70%, and specificity was 70%. Additionally, ESM-1 levels showed positive correlation with CRP and ESR in patients with bone tumors.

The 100% sensitivity and specificity values achieved in distinguishing malignancy with an AUC = 1.000 are noteworthy; however, excellent performance at this level is extremely rare in real-world clinical settings. Due to the limited sample size and the single-center nature of the study, these results may represent overfitting. The relatively small sample size and the fact that the data were obtained from a single center may also limit the generalizability of the findings. Furthermore, the heterogeneous nature of bone tumors limits specific assessments of subtypes. However, multicenter studies with larger sample sizes conducted in different populations and focusing on specific, homogeneous bone tumor subtypes are needed.

One of the most important strengths of this study is that it demonstrates the potential clinical applicability of serum ESM-1 as a minimally invasive and relatively simple biomarker that can be assessed from a peripheral blood sample. However, a significant limitation of the study is the lack of a direct assessment of the relationship between ESM-1 levels and histopathological diagnosis. Since histopathology is the reference method for definitive diagnosis in bone tumors, evaluating the diagnostic performance of ESM-1, particularly its value in differentiating benign and malignant tumors, in conjunction with histopathological findings would provide stronger evidence. Another limitation is the lack of an independent and multicenter validation cohort. In this study, the diagnostic performance and optimal threshold values of ESM-1 were evaluated in a single-center population, and external validation of the findings in larger and independent multicenter cohorts is necessary. Nevertheless, the current findings regarding the potential diagnostic value of serum ESM-1 in bone tumors can provide a basis for future studies in different centers and populations that include direct comparisons with histopathological findings.

## Conclusion

ESM-1 levels may have potential diagnostic value in distinguishing both the presence and malignancy of bone tumors; however, given the limited sample size, single-center nature of the study, heterogeneity of bone tumors, and the possibility of overfitting, these results need to be confirmed by multicenter studies in larger, more homogeneous populations.

## Data Availability

The raw data supporting the conclusions of this article will be made available by the authors, without undue reservation.
